# Acute Effects of Open Kinetic Chain Exercise Versus Those of Closed Kinetic Chain Exercise on Quadriceps Muscle Thickness in Healthy Adults

**DOI:** 10.3390/ijerph17134669

**Published:** 2020-06-29

**Authors:** Soul Cheon, Joo-Hyun Lee, Hyung-Pil Jun, Yong Woo An, Eunwook Chang

**Affiliations:** 1 Department of Kinesiology, Inha University, Incheon 22212, Korea; soul9879@gmail.com (S.C.); joohyun09@gmail.com (J.-H.L.); 2 Institute of Sports & Arts Convergence (ISAC), Inha University, Incheon 22212, Korea; 3 Department of Physical Education, Dong-A University, Busan 49236, Korea; hjun@dau.ac.kr; 4 Department of Health and Human Sciences, Loyola Marymount University, Los Angeles, CA 90045, USA; anyong0047@gmail.com

**Keywords:** quadriceps atrophy, vastus intermedius, vastus medialis, vastus medialis oblique, muscle hypertrophy, resistance exercise, type of exercise

## Abstract

This study aimed to compare immediate changes in the thickness of the rectus femoris (RF), vastus intermedius (VI), vastus lateralis (VL), vastus medialis (VM), and vastus medialis oblique (VMO) muscles after open kinetic chain exercise (OKCE) and closed kinetic chain exercise (CKCE) and identify the effect of both exercise types on each quadricep muscle for early rehabilitation to prevent knee joint injury. Twenty-six healthy participants (13 males and 13 females) were randomly divided into the OKCE (*n* = 13) and CKCE (*n* = 13) groups. The thickness of their quadriceps muscles was measured using a portable ultrasonic imaging device before and after exercise in the sequence RF, VI, VL, VM, and VMO. A two-way repeated measures analysis of variance was used to compare the thickness of each component of the quadriceps muscles between the two groups. The thickness of the RF, VL, VM, and VMO muscles increased after OKCE, and the thickness of the VI muscle showed the greatest increase with a medium–large effect size (F = 8.52, *p* = 0.01, and d = 0.53). The thickness of the VI, VL, VM, and VMO muscles increased after CKCE, and the VMO muscle had the largest effect size (F = 11.71, *p* = 0.00, and d = 1.02). These results indicate that the thickness of the quadriceps muscles can be selectively improved depending on the type of exercise.

## 1. Introduction

The quadriceps femoris muscle belongs to the primary muscle group that is involved in the function of the knee joint. Various sports injuries could result in altered quadriceps characteristics, such as muscle strength, activation, mass, and size of the quadriceps [1,2,3,4]. Previous studies have reported weakness and atrophy of quadriceps muscles after knee joint injuries [2,5]. Recovery of these muscles to their pre-injury state is needed to restore the function of the knee joint [6,7,8]. The quadriceps femoris muscle is made up of five specific muscles—the rectus femoris (RF), vastus intermedius (VI), vastus lateralis (VL), vastus medialis (VM), and vastus medialis oblique (VMO). Although this group of muscles normally functions as a knee extensor, previous studies have reported a specific function of each muscle component. The RF is a biarticular muscle that connects the hip joint to the knee joint and acts as a primary knee muscle extensor; 33% force is exerted while bending the hip joint [9]. The VM is a primary knee extensor muscle, and the VMO muscle acts as a medial stabilizer of the patella [10]. Although the VMO muscle is weaker than the VL muscle, it controls the lateral deviation of the patella [11,12]. The imbalance of forces between the VM and VL muscles results in patellar instability, causing movement dysfunction and inducing patella femoral pain syndrome (PFPS) [13]. Each quadriceps muscle has a specific role in movement; hence, it is necessary to investigate the characteristic of each quadricep muscle for functional improvement.

Open kinetic chain exercise (OKCE) and closed kinetic chain exercise (CKCE) are the types of exercise based on the fixed point of the extremity during movements. Although they have been used in the clinical field, each exercise has a specific purpose and characteristics. OKCE is considered less functional than CKCE, but it plays an important role in improving muscle strength during rehabilitation in patients with a limited range of motion [14]. Additionally, it improves the muscle strength of each quadricep muscle or the entire muscle without compensating the movement [15]. CKCE can be performed by applying varying ranges of motion with a functional speed. This type of exercise requires action of the antagonistic muscles to eccentrically control the movements by providing stability to the damaged joints [16]. Therefore, CKCE has been recommended in the early stages of rehabilitation after the anterior cruciate ligament reconstruction (ACLR) [14].

In previous studies evaluating the effects of OKCE and CKCE on the quadriceps femoris muscle [14,17,18], the activity of the RF muscle increased by 45% after OKCE compared to that after CKCE [14]. In another study, OKCE was effective for the activation of the quadriceps muscles during the first two weeks of rehabilitation [17] and restoring the ratio of the VM muscle to the VL muscle after knee joint surgery [18]. In a recent study, muscle thickness was considered a significant factor for identifying muscle strength and knee extension torque, and joint functions were predicted by the thickness of the VI and VMO muscles after ACLR [19]. While these previous investigations revealed the different effects on quadriceps activity by exercise type and the importance of muscle thickness, the acute effect on the thickness of each quadricep muscle after OKCE and CKCE was not investigated. Therefore, this study aimed to compare the acute effect of OKCE and CKCE on the thickness of each quadricep muscle. We hypothesized that there is a significant difference in the thickness of the quadricep femoris muscles before and after OKCE and CKCE.

## 2. Materials and Methods

### 2.1. Participants

Twenty-six healthy adults (13 males and 13 females; age: 24.3 ± 3.8 years; height: 169.3 ± 7.2 cm; weight: 66.4 ± 12.9 kg; and body mass index (BMI) [17]: 23 ± 3.6 kg/m^2^) who participated in this study were randomly divided into the OKCE (*n* = 13; male: 8, female: 5) and CKCE (*n* = 13; male: 7, female: 6) groups. Participants having current knee joint pain and those who had undergone surgeries of the lower back and extremities in the past 6 months were excluded from the study. The general characteristics of the study participants are shown in Table 1. All participants signed an informed consent form after understanding the purpose of the study. All procedures were approved by the University’s Institutional Review Board (Study ID: 190404).

### 2.2. Study Design

**Figure 1:**
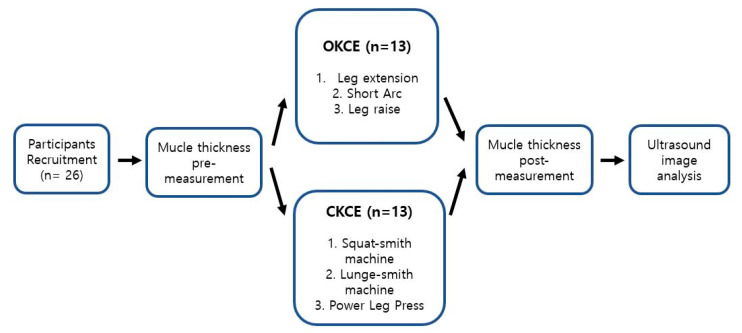
Overview of the study design, demonstrating the muscle thickness pre- and post-measurement.

The study design is presented in Figure 1. All participants visited the laboratory for a day during the period of the study. Before the exercises, the thickness of the quadriceps muscles was measured using a portable B-mode ultrasound device (Healcerion, Seoul, Korea) with a linear-array transducer (7.5 MHz). The ultrasound device was set for gain (dB), depth (5 cm), and frequency (12 MHz) for all images. Each participant in the OKCE and CKCE groups performed three exercises and the thickness of their quadriceps muscles was measured in the same position immediately after the exercises.

### 2.3. Experimental Methods

#### 2.3.1. Muscular Thickness Measurement

**Figure 2:**
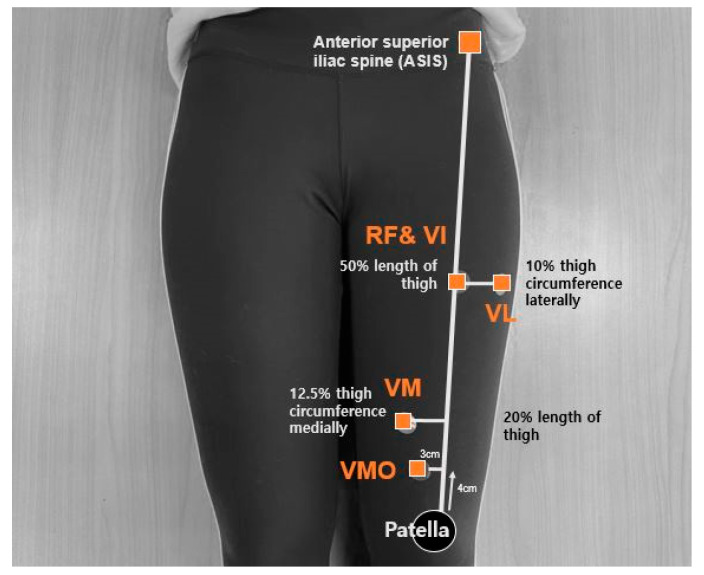
Location of quadriceps thickness measurement. RF: rectus femoris; VI: vastus intermedius; VL: vastus lateralis; VM: vastus medialis; VMO: vastus medialis oblique.

Ultrasound images were obtained using a portable ultrasonic imaging device and a portable tablet personal computer (IPad, Foxconn, Taipei, Taiwan). All muscle thickness measurements were performed by one investigator. Previous investigations reported excellent intra-rater (Intraclass Correlation Coefficients (ICCs) 0.95–0.97) and acceptably good inter-rater (ICCs 0.62–0.90) reliability of the quadriceps thickness measurements using ultrasound [20,21]. To measure the thickness of each quadricep muscle, the participants were made to lie in a supine position to prevent external hip rotation. The leg of the participants that was farthest away from the ball was considered the dominant leg [22]. The dominant leg of the participants was assessed after the participants were made to lie in a supine position with both legs fully extended for 10 min to stabilize the fluid shifts [23]. A water-soluble gel that was applied between the transducer and skin enhanced the acoustic contact and reduced the risk of image misinterpretation [24]. The transversal images of the quadriceps muscles were acquired in the sequence RF, VI, VL, VM, and VMO. To obtain the images, a virtual line was marked along the length of the thigh from the superior pole of the patella to the anterior superior iliac spine (ASIS). The RF and VI were measured on and at 50% of the virtual line. In order to measure VL, thigh circumference at 50% of the virtual line was measured, and then VL was measured as 10% of the measured circumference distance laterally from the virtual line. The VM muscle was measured at 20% distance of the virtual line, and then the circumference was measured on the same point. Once the measurement of the circumference was completed, VM was measured at 12.5% of the measured circumference distance medially from the virtual line [25]. The VMO muscle was measured 4 cm superior and 3 cm medial of the superior pole of the patella (Figure 2) [26]. The gain was adjusted until the femur was in the center of the screen such that the boundary of the muscle was visible, and then the depth of the image was measured. Images of each muscle were recorded three times. The ultrasound images were saved for further analysis after the muscle thickness was measured.

#### 2.3.2. Exercise

The National Strength and Conditioning Association standard states that 3–6 sets of an exercise at 6–12 repeated maximums (RM: 67–75% of 1 RM) is effective for muscle hypertrophy [27]. Therefore, the intensity of the exercises was set at 10 RM. The participants performed three sets of exercises with 10 repetitions each, with a 60-s rest between sets [28].

#### 2.3.3. Open Kinetic Chain Exercise

**Figure 3:**
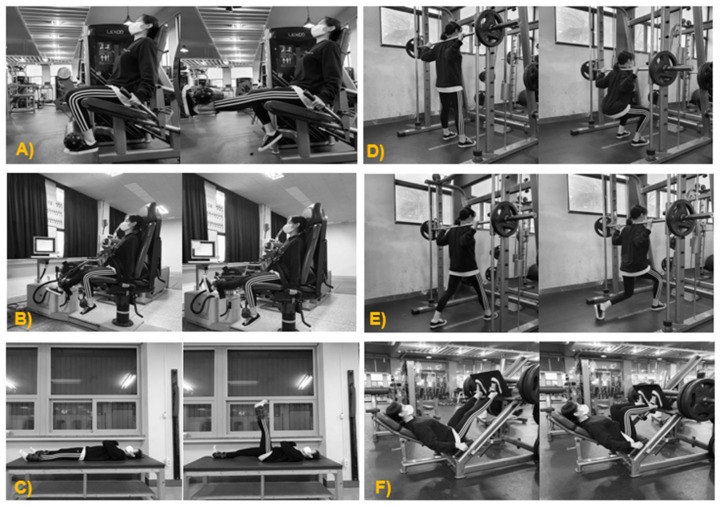
OKCE and CKCE exercise. OKCE: (**A**) leg extension; (**B**) short-arc; (**C**) leg raise. CKCE: (**D**) squat—Smith machine; (**E**) lunge—Smith machine; (**F**) leg press.

OKCE consisted of leg extension, short-arc, and straight leg raise exercises. Leg extension and short-arc exercises were performed using an exercise machine and straight leg raise exercise was performed as a free-weight exercise (Figure 3).

1. Leg extension exercise
  - Participants were positioned on the center of the back of a machine such that their thighs, backs, and heads were not tilted on one side. Their knees were aligned in a straight line along the axis of the machine and their hips and thighs were positioned such that the back of their knees touched the edge of the chair. The starting position was set at a flexion of 90°. The knees of the participants were not in hyperextension or hyperflexion during the exercise and their upper body was stationary [27].
2. Short-arc exercise
  - The short-arc exercise was performed using a knee extension exercise machine. Although the participant was positioned in the same way as that of the leg extension exercise, the starting position of the knee was at a flexion of 20°. Participants performed knee extensions and a hold of 2–3 s in the fully extended position.
3. Leg Raises Exercise
  - Sandbags with a predetermined weight of 10 RM were attached to ankle of each of the participant. They initiated a straight leg raise while lying on the table with both hands on their chest and one leg extended. The other leg was stabilized at the knee flexed with 90°.

#### 2.3.4. Closed Kinetic Chain Exercise

The participants in the CKCE group performed three exercises—squat, lunge, and leg press. Squat and lunge exercises were performed using a Smith machine and the leg press exercise was performed on a linear 45° leg press machine (Figure 3).

1. Squat—Smith machine
  - Participants were made to stand while they held a bar in pronation grip across their shoulders. Their feet were positioned at a slightly wider distance than the shoulders and toes were pointed slightly outside. The participants initiated the exercise in the squat position while slowly flexing their hips and knees and keeping the body angle constant [27].
2. Lunge—Smith machine
  - Participants were made to stand and a bar was placed above the posterior deltoid and upper trapezius in pronation grip that was wider than their shoulder. They maintained a straight posture and one foot was placed forward and the other was placed behind. Their front leg was horizontal to the floor and their rear leg was vertical to the floor, while the knees and hips of their front legs were bent gradually. They were instructed not to bend the front knee past the front foot and not to bend the trunk forward. The trial was repeated if this requirement was not met [27].
3. Leg press machine
  - Participants were positioned on the center of the back of a machine such that their thigh, back, and head were not tilted on one side and they held the handle on both sides. Their knees were extended and their feet were positioned above the footpad within shoulder width. Participants were seated at an angle of approximately 120°. The knee of each participant extended and protracted back to the starting position. The heels of the participants remained on the footpads and the knees were not in hyperextension or hyperflexion. The upper body of the participants remained stationary [27].

#### 2.3.5. Ultrasound Image Analysis

**Figure 4:**
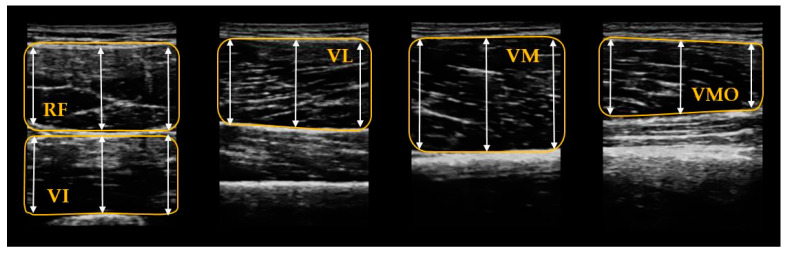
Image of the thickness of the quadriceps muscles. RF: rectus femoris; VI: vastus intermedius; VL: vastus lateralis; VM: vastus medialis; VMO: vastus medialis oblique.

The recorded ultrasound images were analyzed using Image J software (National Institute for Health, Bethesda, MD, USA). Each image was scaled individually for converting an area in pixels to centimeters using the straight-line function to analyze the muscle thickness [29]. According to previous studies, the region of interest within each muscle was selected, excluding the surrounding bone and fascia [30,31,32]. Muscle thickness was defined as the widest distance between the adipose muscle upper interface and the lower interface for all quadriceps muscles, excluding the VI muscle [33]. The VI muscle was measured as the widest distance between the adipose muscle upper interface and the femur (Figure 4) [33]. One middle line and two lines were placed at regular intervals on both sides around middle line and the average values of the three lines were obtained [34]. The images were analyzed by an investigator who was unable to see the participants’ identity. The average values of the three images per muscle were statistically analyzed.

### 2.4. Statistical Analysis

Demographic characteristics of the participants were compared between the two groups using an independent t-test. Continuous variables, such as age, height, mass, and BMI, are presented as the mean ± SD. A two-way repeated measures analysis of variance (ANOVA) (group: OKCE and CKCE; by time: pre-exercise and post-exercise) compared the thickness of each quadricep muscle before and after the exercise. A post hoc analysis was performed using the Tuckey method for a group-by-time interaction effect. Cohen’s d effect size was calculated to determine the magnitude of change in muscle thickness before and after exercise (small = 0.2–0.49, medium = 0.5–0.79, and large > 0.8) [35]. All data are expressed as the mean and SD. Statistical analyses were performed using SPSS 25.0 software (SPSS Inc., Chicago, IL, USA). A *p*-value < 0.05 was considered statistically significant.

## 3. Results

For the general characteristics, a significant statistical difference was not observed (*p* > 0.05) between the groups (Table 1).

**Figure 5:**
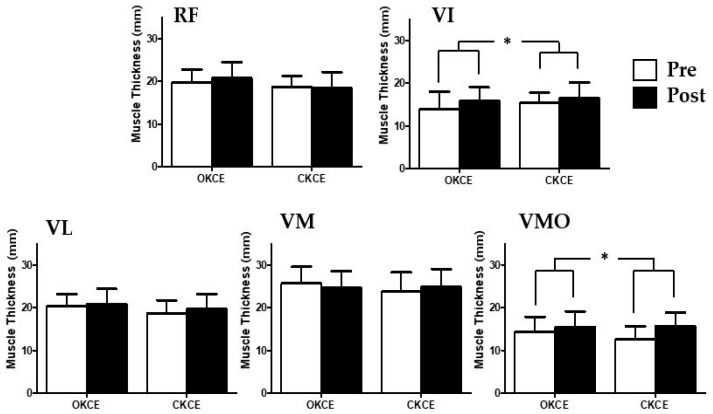
Comparison of the changes in quadricep muscle thickness. OKCE: open kinetic chain exercise, CKCE: closed kinetic chain exercise, RF: rectus femoris; VI: vastus intermedius; VL: vastus lateralis; VM: vastus medialis; VMO: vastus medialis oblique. * *p* < 0.05 indicates a significant difference between pre- and post-intervention within the group.

The thickness of the VI muscle increased in both groups (OKCE pre: 13.92 ± 4.08 mm, post: 15.86 ± 3.13 mm; CKCE pre: 15.3 ± 2.46 mm, post: 16.47 ± 3.66 mm). For the VI muscle, there was a significant time effect in the thickness (F = 8.52, *p* = 0.01) and its effect size was greater for OKCE (d = 0.53) than CKCE (d = 0.38) (Table 2) (Figure 4). The thickness of the VMO muscle was increased in both groups (OKCE pre: 14.37 ± 3.50 mm, post: 15.42 ± 3.70 mm; CKCE pre: 12.59 ± 2.96 mm, post: 15.72 ± 3.19 mm). For VMO muscle, there was a significant time effect in the thickness only (F = 11.71, *p* = 0.00). While CKCE exhibited a large effect size (d = 1.02), OKCE showed a small effect size (d = 0.29) (Table 2) (Figure 5). Finally, there was no significant time-by-group interaction, group, and time main effect on RF, VL, and VM (*p* > 0.05) (Table 2 and Figure 5).

## 4. Discussion

This study aimed to compare the effects of OKCE and CKCE on the thickness of each quadricep muscle. Previous studies have reported that many patients with lower extremity injuries developed quadriceps atrophy after surgery or injury [36,37]. Since each quadricep muscle has different functions, the patterns of atrophy are different [19,38,39]. Therefore, it is important to identify an appropriate method of exercise for each quadricep muscle. In this study, the thickness of the VI muscle increased significantly after OKCE, and the thickness of the VMO muscle increased significantly after CKCE, resulting in the medium–large effect size. These findings suggest that the effects of OKCE and CKCE in improving the overall thickness of the quadriceps muscles, especially in early rehabilitation training, is appropriate for patients with ACLR or PFPS who need selective strengthening of the VI and VMO muscles.

According to a previous study, the VMO and VL are the principle muscles that stabilize the patella during dynamic knee extension [11]. Although the ideal ratio of the EMG activity of the VMO-to-VL for healthy people is 1:1 [12], patients with PFPS in a previous study had a ratio of 0.54:1, resulting in a weakening of the VMO muscle [40]. In another previous study, the ultrasonographic measurements of the VMO muscle were smaller in individuals with PFPS knees than in healthy people [41]; this imbalance between the two muscles resulted in an abnormal tracking of the patella, causing joint pain [42]. Therefore, rehabilitation exercises are important to strengthen the VMO muscles in such patients. In the present study, a large effect size was observed in the thickness of the VMO muscle after CKCE.

In the current study, three CKCEs were performed, including squat, lunge, and leg press; previous investigations reported that these exercises were naturally accompanied by hip adduction with knee joint movement [11,43]. These findings support the results of a prior study, in which CKCE with isometric hip adduction significantly improved the ratio of VMO muscle to VL muscle compared to OKCE, where only knee joints were involved independently [11]. An anatomic cadaver study has suggested the origin of the VMO muscle from the distal part of the adductor magnus and has revealed that the VMO muscle exerts a greater force after CKCE than OKCE [44]. Therefore, CKCE could be useful during the initial stage of rehabilitation of patients with PFPS.

The VI muscle acts on knee extension torque and is essential for athletes who need explosive movements [19]. A previous study has found a reduction in the thickness of the VI muscle in patients with ACLR and has suggested that increasing this thickness during the early phases of rehabilitation is a key factor for restoring the knee function [45]. In a prior study, the range of motion of the knee joint was limited in the early stages of the knee rehabilitation program; hence, it suggests that partial muscle strengthening exercises should be performed along with OKCE [18]. In the present study, the thickness of the VI muscles showed medium–large effect sizes after OKCE compared to that after CKCE. OKCE can be a more effective rehabilitation exercise than CKCE to restore the thickness of the VI muscle after ACLR.

It could be interesting that OKCE showed the greatest influence in increasing VI thickness compared to the other quadricep muscle components. VI is the quadriceps component that originate from the femur and it functions in knee extension only, whereas another component helps for hip flexion [46]. Therefore, it could be speculated that the independent movement of the knee joint, such as knee extension during OKCE, requires the greatest efforts on VI. Additionally, VI exhibited the highest estimated torque contribution during isometric knee extension among the quadriceps components [47], which supports the results of the current study.

Both exercises were able to improve the thickness of the quadriceps muscles. Further, proper methods can be used to effectively exercise the individual muscles for improvement. Hooper et al. (2001) [48] and Perry et al. (2005) [49] have reported that although CKCE is more effective in improving functional movements than OKCE, there is no significant difference when they were measured by actual clinical evaluation indicators in patients with ACLR. However, sophisticated research is needed because the results vary depending on the subjects or methods for evaluating the functions and errors. The study has some limitations. First, it was not possible to ascertain whether each participant performed the exercise effectively. Secondly, although both men and women participated in this study, sex-related hormones were not checked. Thirdly, the sample size was small and the adults were healthy. Therefore, further studies enrolling participants of different age groups and patients with various lower-limb injuries are needed to generalize the current study results. Fourthly, exercise selection was decided based on the feasibility of the clinical setting and it was not controlled. Finally, we obtained an acute effect, not a long-term effect, after the intervention. Further studies are needed to compare the muscle reactions before and after exercise as well as between acute and chronic exercise.

## 5. Conclusions

This study was conducted to investigate the acute effects of OKCE and CKCE on the thickness of the five individual quadriceps muscles. The thickness of the VI muscle improved after OKCE and the thickness of the VMO muscle increased significantly after CKCE. The findings of this study can help athletes and patients with knee joint injuries choose the most appropriate exercise during the early stages of rehabilitation. OKCE and CKCE should be properly used for selective strengthening of the quadriceps muscles.

## Figures and Tables

**Table 1:** General characteristics of the study participants.

Characteristics	OKCE (*n* = 13)	CKCE (*n* = 13)	*p*-Value
Age (years)	23.5 ± 1.7	25.1 ± 5.1	0.30
Height (cm)	170.2 ± 6.5	168.4 ± 8.0	0.53
Mass (kg)	67.8 ± 15.6	65.1 ± 9.8	0.61
BMI (kg/m^2^)	23.2 ± 4.6	22.9 ± 2.5	0.82

Values are presented as the mean ± standard deviation (SD). OKCE: open kinetic chain exercise; CKCE: closed kinetic chain exercise; BMI: body mass index.

**Table 2:** Comparison of the quadriceps muscle thickness between the groups pre- and post-intervention.

Muscles	OKCE			CKCE			*p*-Value
	Pre	Post	ES	Pre	Post	ES	
RF(mm)	19.72 ± 2.98	20.70 ± 3.70	0.29	18.71 ± 2.47	18.45 ± 3.68	−0.08	0.59
VI(mm)	13.92 ± 4.08	15.86 ± 3.13	0.53	15.30 ± 2.46	16.47 ± 3.66	0.38	* 0.01
VL(mm)	20.39 ± 2.77	20.87 ± 3.46	0.15	18.68 ± 3.00	19.77 ± 3.36	0.34	0.20
VM(mm)	25.61 ± 3.92	24.58 ± 3.95	−0.26	23.71 ± 4.51	24.83 ± 4.20	0.26	0.98
VMO(mm)	14.37 ± 3.50	15.42 ± 3.70	0.29	12.59 ± 2.96	15.72 ± 3.19	1.02	* 0.00

Values are presented as the mean ± SD. ES: Cohen’s d effect size; OKCE: open kinetic chain exercise; CKCE: closed kinetic chain exercise; RF: rectus femoris; VI: vastus intermedius; VL: vastus lateralis; VM: vastus medialis; VMO: vastus medialis oblique. * *p* < 0.05 indicates a significant difference between pre- and post-intervention within the group.
